# PRELP functions via multiple interactions with intrinsically weak affinity relying on ECM anchoring and remodeling

**DOI:** 10.1038/s41598-025-09018-7

**Published:** 2025-07-09

**Authors:** Hirofumi Kosuge, Makoto Nakakido, Susana de Vega, Shin-ichi Ohnuma, Kouhei Tsumoto

**Affiliations:** 1 Department of Bioengineering, School of Engineering, The University of Tokyo, 7- 3-1, Hongo, Bunkyo-ku, Tokyo, 113-8656 Japan; 2 Department of Chemistry and Biotechnology, School of Engineering, The University of Tokyo, 7-3-1, Hongo, Bunkyo-ku, Tokyo, 113-8656 Japan; 3 The Institute of Medical Science, The University of Tokyo, 4-6-1, Shirokanedai, Minato-ku, Tokyo, 108-8639 Japan; 4 The Institute of Ophthalmology, University College London, London, EC1V 9EL UK

**Keywords:** Biochemistry, Biochemistry, Biophysics, Cell biology

## Abstract

A small leucine-rich repeat proteoglycan PRELP is responsible for various biological functions. Here, to quantitatively assess the ligand binding of PRELP and its relevance to physiological activities, we validated the premise that PRELP multi-specifically binds to TGFβ1, IGFI-R, and p75NTR with relatively weak, micromolar range of affinities using surface plasmon resonance analysis. Results of a direct binding assay using N-terminal-truncated PRELP and chimeric PRELP and a dual injection assay to evaluate the binding regions and competitiveness suggested that PRELP interacts with the ligands via different but partially overlapping regions in the leucine-rich repeat domain. RNA-seq analysis revealed that PRELP greatly promotes gene expression of various extracellular matrix (ECM) components in A549 lung carcinoma cells, also at micromolar concentration. Since we reasoned that ECM anchoring contributes to an increase of apparent local concentrations of PRELP required for the weak affinity interactions, we validated the direct binding and co-localization of PRELP with ECM proteins using ELISA analysis and immunofluorescence staining. Results of this study suggest that PRELP modulates multiple interactions with intrinsically weak binding affinities through the anchoring to ECM proteins and also promotes the ECM protein expression to maintain the preferred environment to exert the molecular functions.

**Supplementary Information:**

The online version contains supplementary material available at 10.1038/s41598-025-09018-7.

## Introduction

The small leucine-rich repeat proteoglycan (SLRP) family consists of 18 secreted proteins with diverse biological functions that are known to be localized in the extracellular matrix (ECM)^1–3^. In the ECM environment, many of these proteins bind to different types of collagen fibrils, leading to the regulation of collagen fibril growth and ECM assembly^4,5^. Many SLRPs also regulate transduction of multiple signals through their interactions with a variety of cytokines and cell-surface receptors^1,3^. Given that SLRPs would exert diverse molecular functions through the multiple ligand binding, quantitative clarification of the molecular basis for the multi-specific interactions of SLRPs should be helpful to better understand the biological functions of SLRPs.

Proline/arginine-rich end leucine-rich repeat protein (PRELP) is a member of class II SLRPs, and it is known to exert diverse biological functions relying on the interactions with various proteins including complement components^6^ and ECM proteins^7,8^. PRELP also has tumor suppressive roles^9–13^, and expression of *PRELP* is down-regulated in a wide variety of cancer types^13–15^. Given that recent studies revealed that PRELP interacts with several growth factors including transforming growth factor β1 (TGFβ1) involved in signal transduction in cancer^11,13^, PRELP is expected to suppress tumor cell growth triggered by the multi-specific ligand binding. In this sense, it has been revealed that PRELP interacts with TGFβ1, leading to the suppression of canonical SMAD signal transduction^13,16^ and suggested that PRELP suppresses bladder cancer progression by reversing the epithelial-mesenchymal transition through inhibition of TGFβ1 signaling pathway^13^. Previously, we also identified two growth factor receptors, insulin-like growth factor I receptor (IGFI-R) and low-affinity nerve growth factor receptor (p75NTR), as binding ligands for PRELP and illustrated that the interaction affinity was in the micromolar range. We further showed that PRELP at micromolar concentrations suppresses cancer cell proliferation, which is in agreement with the binding affinity for IGFI-R and p75NTR^17^. These observations highlighted the importance of quantitative evaluation of interactions with each binding partner, which is difficult to assess by genetic-based approaches, to understand the pleiotropic molecular functions of PRELP.

In this study, we used surface plasmon resonance (SPR) analysis to validate the direct binding and measure the binding affinities of PRELP to multiple ligands including TGFβ1, TGFβ type II receptor (TGFβRII), IGFI-R, and p75NTR and investigated the binding competition between these ligands through a binding assay using N-terminal-deleted PRELP and chimeric PRELP constructs and a dual injection assay. To quantitatively evaluate the physiological activity of PRELP, we also analyzed the alteration of gene expression levels in A549 lung carcinoma cells triggered by the addition of recombinant PRELP and showed significant up-regulation of various ECM protein expression at micromolar PRELP concentration. Furthermore, we conducted ELISA binding analyses and cell immunofluorescence assays to study how PRELP protein accomplishes its functions, which appear to require relatively high, micromolar range of concentrations, in the extracellular environment and showed the direct-binding and co-localization of PRELP with ECM proteins. These results suggest that the apparent local concentration of PRELP is enhanced by anchoring to ECM components, enabling PRELP to interact with binding targets including TGFβ1, IGFI-R, and p75NTR at the proximity of cell surface in spite of intrinsic weak affinities, and moreover, PRELP appears to enhance ECM organization to maintain the preferred environment to exert the molecular functions.

## Results

### Quantitative evaluation of interactions between PRELP and multiple ligands

**Fig. 1:**
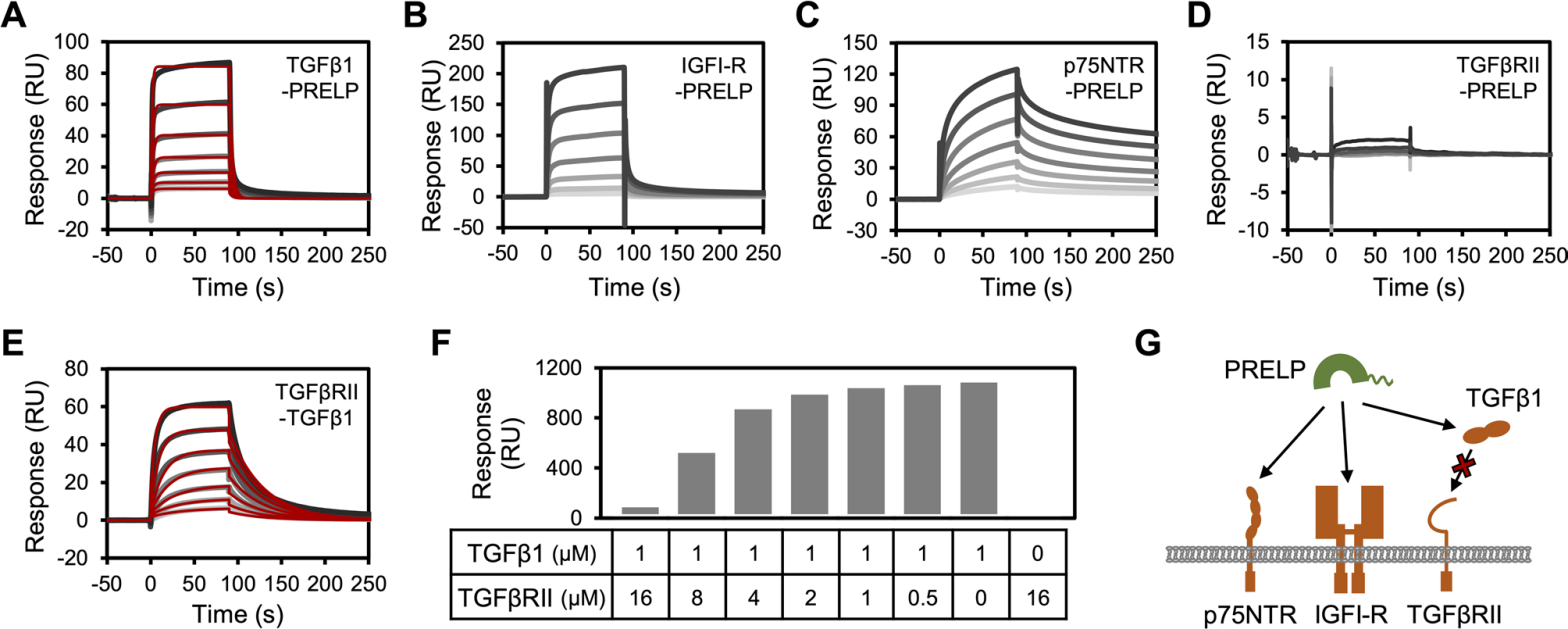
Interaction analysis between PRELP and multiple ligands. (**A**–**D**) Direct binding assay of immobilized PRELP with (**A**) TGFβ1 (15.6–1000 nM), (**B**) IGFI-R (312.5–20000 nM), (**C**) p75NTR (625–40000 nM), and (**D**) TGFβRII (156–10000 nM) using SPR. The raw sensorgrams and curve fitting profiles are shown as monochrome and red lines, respectively. Line darkness of raw sensorgrams indicates analyte concentrations. The darkest line indicates the highest concentration. (**E**) Direct binding assay using SPR between immobilized TGFβ1 and TGFβRII (15.6–1000 nM). (**F**) Competitive binding analysis. A mix of TGFβ1 with a range of concentrations of TGFβRII was injected into PRELP immobilized on a sensor chip. (**G**) Model of pleiotropic regulation of ligand interactions by PRELP.

To verify the interaction of PRELP with TGFβ1, TGFβRII, IGFI-R, and p75NTR, a growth factor and growth factor receptors which are indicated as binding ligands of PRELP in previous studies^11,13,16,17^, we prepared recombinant proteins (Supplementary Fig. S1) and quantitatively analyzed the direct binding using SPR. The results showed that PRELP directly bound to TGFβ1 with a submicromolar affinity (Fig. 1A; Table 1). We also conducted SPR analysis at a range of temperatures, and thermodynamic parameters calculated from van’t Hoff plots indicated an enthalpy-driven interaction between PRELP and TGFβ1 (Supplementary Fig. S2, Supplementary Table S1), suggesting specific binding dominated by electrostatic interactions and hydrogen bonds. The interactions of PRELP with IGFI-R and p75NTR ectodomains were also measured using SPR, and dissociation constants were calculated by equilibrium curve fitting (Figs. 1B–C, Supplementary Fig. S3). PRELP showed micromolar range of binding affinities for IGFI-R and p75NTR (Table 1) in agreement with our previous study using a different Biacore system^17^. In contrast, a significant binding response deserving evaluation of binding affinity was not observed for TGFβRII ectodomain and PRELP (Fig. 1D) although TGFβRII interacted with TGFβ1 with a submicromolar affinity (Fig. 1E; Table 1), which is consistent with results of a previous study^18^. Because recent studies showed that PRELP interacted with TGFβ1 and inhibited the signal transduction^13,16^, PRELP is expected to impede the interaction between TGFβ1 and TGFβRII. To address this hypothesis, competitive binding analysis was performed using SPR. A mix of TGFβ1 with different concentrations of TGFβRII was injected into PRELP immobilized on a sensor chip, and the binding response was evaluated. The competitive binding analysis showed that TGFβRII inhibited the interaction between PRELP and TGFβ1 in a concentration-dependent manner (Fig. 1F, Supplementary Fig. S4). These results indicate that PRELP multi-specifically binds to TGFβ1, IGFI-R, and p75NTR with relatively weak binding affinities (*K*_D_ = 10^− 7^ ~ 10^− 5^ M) and suggest PRELP-mediated inhibition of TGFβ1-TGFβRII interactions (Fig. 1G).

**Table 1:** Kinetic parameters of the interaction of PRELP with multiple ligands.

	*k*_on_ (× 10^5^ M^–1^ s^–1^)	*k*_off_ (s^–1^)	*K*_D_ (× 10^–6^ M)
PRELP—TGFβ1	5.13 ± 0.28	0.196 ± 0.023	0.380 ± 0.037
PRELP—IGFI-R	N.D	N.D	7.09 ± 1.13
PRELP—p75NTR	N.D	N.D	12.7 ± 2.5
TGFβ1—TGFβRII	1.65 ± 0.29	0.0301 ± 0.0037	0.189 ± 0.012

At least three independent measurements were carried out. The average values with standard errors are shown. *k*_on_, *k*_off_, and *K*_D_ represent association rate constant, dissociation rate constant, and dissociation constant, respectively. N.D. indicates “not determined.”

### Investigation of binding regions using N-terminal-truncated PRELP and chimeric PRELP

**Fig. 2:**
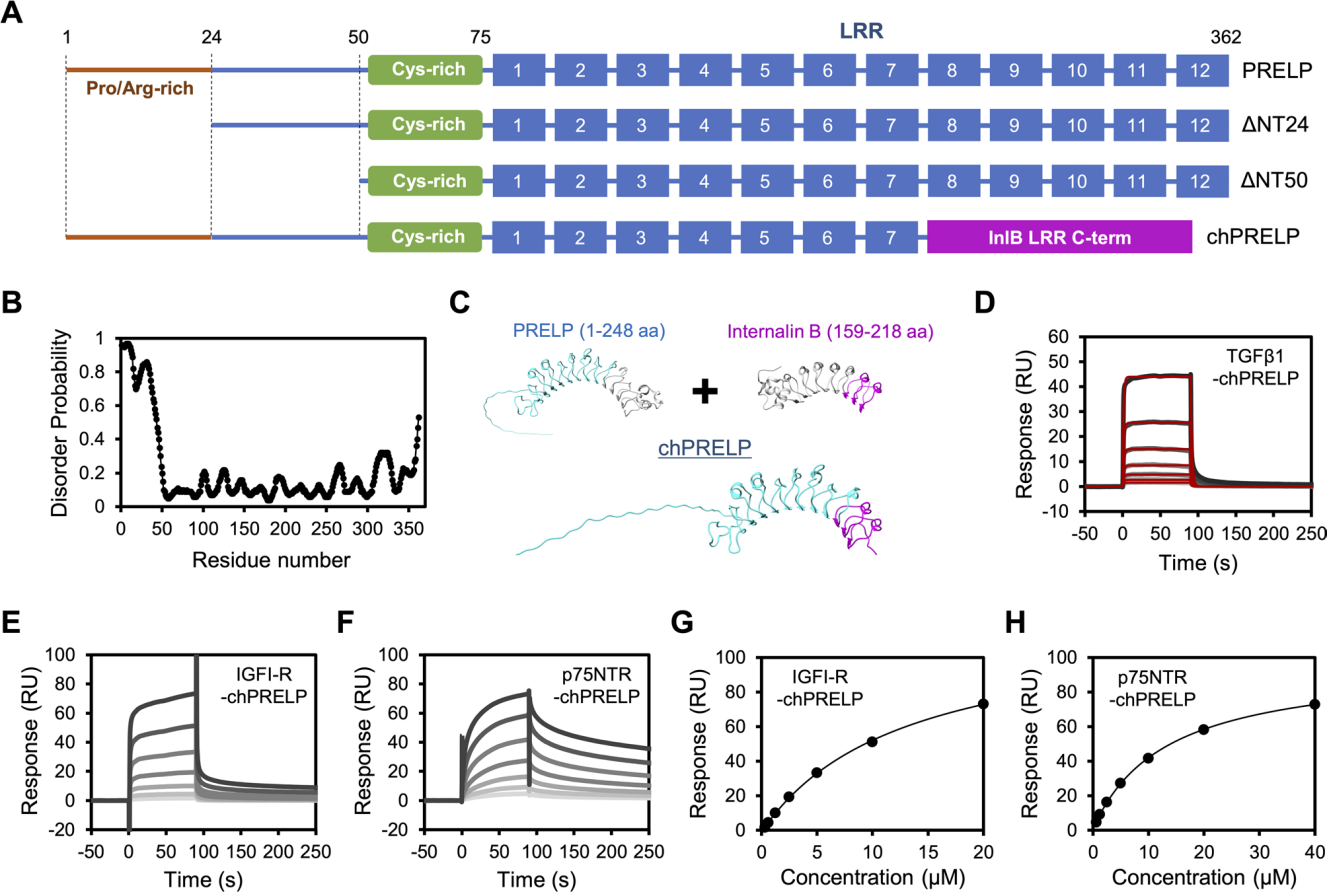
Interaction analysis using N-terminal-truncated PRELP and chimeric PRELP. (**A**) Constructs of ΔNT24, ΔNT50, and chPRELP. (**B**) Disorder probability of PRELP predicted by PrDOS. (**C**) Structural model of the chPRELP. A model PRELP and chPRELP structure was made using Google AlphaFold2_mmseq2 notebook from the ColabFold project. Structural data of Internalin B was from Protein Data Bank (PDB ID: 6DBF). (**D**–**F**) Direct binding assay of immobilized chPRELP with (**D**) TGFβ1 (15.6–1000 nM), (**E**) IGFI-R (312.5–20000 nM), and (**F**) p75NTR (625–40000 nM) using SPR. The raw sensorgrams and curve fitting profiles are shown as monochrome and red lines, respectively. Line darkness of raw sensorgrams indicates analyte concentrations. The darkest line indicates the highest concentration. (**G**–**H**) Response units vs. concentration of (**G**) IGFI-R and (**H**) p75NTR for interaction with chPRELP.

PRELP is composed of a distinctive proline/arginine-rich N-terminal region as well as a core domain consisting of twelve leucine-rich repeats (LRRs), which are shared with other SLRPs (Fig. 2A). PRELP binds to collagen fibrils through the core LRR domain^8^, while the N-terminal proline/arginine-rich part of PRELP plays a crucial role in the interactions with heparin and heparan sulfate^7,8^. The N-terminal region of PRELP was predicted to be intrinsically disordered using PrDOS server^19^ (Fig. 2B), and PRELP has a cysteine-rich cluster between the N-terminal disordered region and LRR domain, which is likely important for the protein structure. Therefore, to identify the binding sites of PRELP that are involved in the interaction with multiple ligands, we prepared recombinant N-terminal-truncated PRELP ΔNT24 and ΔNT50, which lack the proline/arginine-rich region and whole N-terminal region, respectively (Fig. 2A). Circular dichroism (CD) measurements did not show large differences in the secondary structure of N-terminal-truncated PRELP compared with full-length PRELP (Supplementary Fig. S5). The direct binding assay using SPR demonstrated that ΔNT24 and ΔNT50 bound to TGFβ1, IGFI-R, and p75NTR although the binding affinities were a little different compared with full-length PRELP (Supplementary Fig. S6, Tables 2 and 3), indicating that these ligands interact with the LRR domain of PRELP.

**Table 2:** Kinetic parameters of the interaction of N-terminal-truncated PRELP and chimeric PRELP with TGFβ1.

	*k*_on_ (× 10^5^ M^–1^ s^–1^)	*k*_off_ (s^–1^)	*K*_D_ (× 10^–6^ M)
ΔNT24	2.77 ± 0.03	0.142 ± 0.008	0.512 ± 0.028
ΔNT50	0.751 ± 0.295	0.169 ± 0.113	1.55 ± 0.61
chPRELP	2.41 ± 0.49	0.362 ± 0.149	1.21 ± 0.37

At least three independent measurements were carried out. The average values with standard errors are shown. *k*_on_, *k*_off_, and *K*_D_ represent association rate constant, dissociation rate constant, and dissociation constant, respectively.

**Table 3:** Binding affinities of the interaction of N-terminal-truncated PRELP and chimeric PRELP with IGFI-R and p75NTR.

	ΔNT24	ΔNT50	chPRELP
IGFI-R	3.90 ± 1.42	6.97 ± 3.33	15.4 ± 1.4
p75NTR	11.3 ± 3.4	11.7 ± 3.4	14.5 ± 2.1

At least three independent measurements were carried out. The average values of *K*_D_ (µM) calculated by equilibrium curve fitting with standard errors are shown.

To further validate the binding regions, we employed a construct composed of a N-terminal half portion of PRELP and the C-terminal region of the LRR domain of internalin B (InlB) (Fig. 2A), referring to an approach previously reported for other LRR proteins^4,20^. The chimeric PRELP (chPRELP) was designed by sequential and structural homology between PRELP and InlB (Fig. 2C), and the secondary structure was confirmed by CD measurements (Supplementary Fig. S5). The direct binding assay using SPR demonstrated that chPRELP bound to TGFβ1, IGFI-R, and p75NTR (Figs. 2D–H; Tables 2 and 3). The results of interaction analyses using N-terminal-truncated and chimeric PRELP constructs indicate that the LRR1-7 region provides PRELP with the multi-specificity for different ligands.

### Assessment of simultaneous ligand binding to PRELP using dual injection assay

**Fig. 3:**
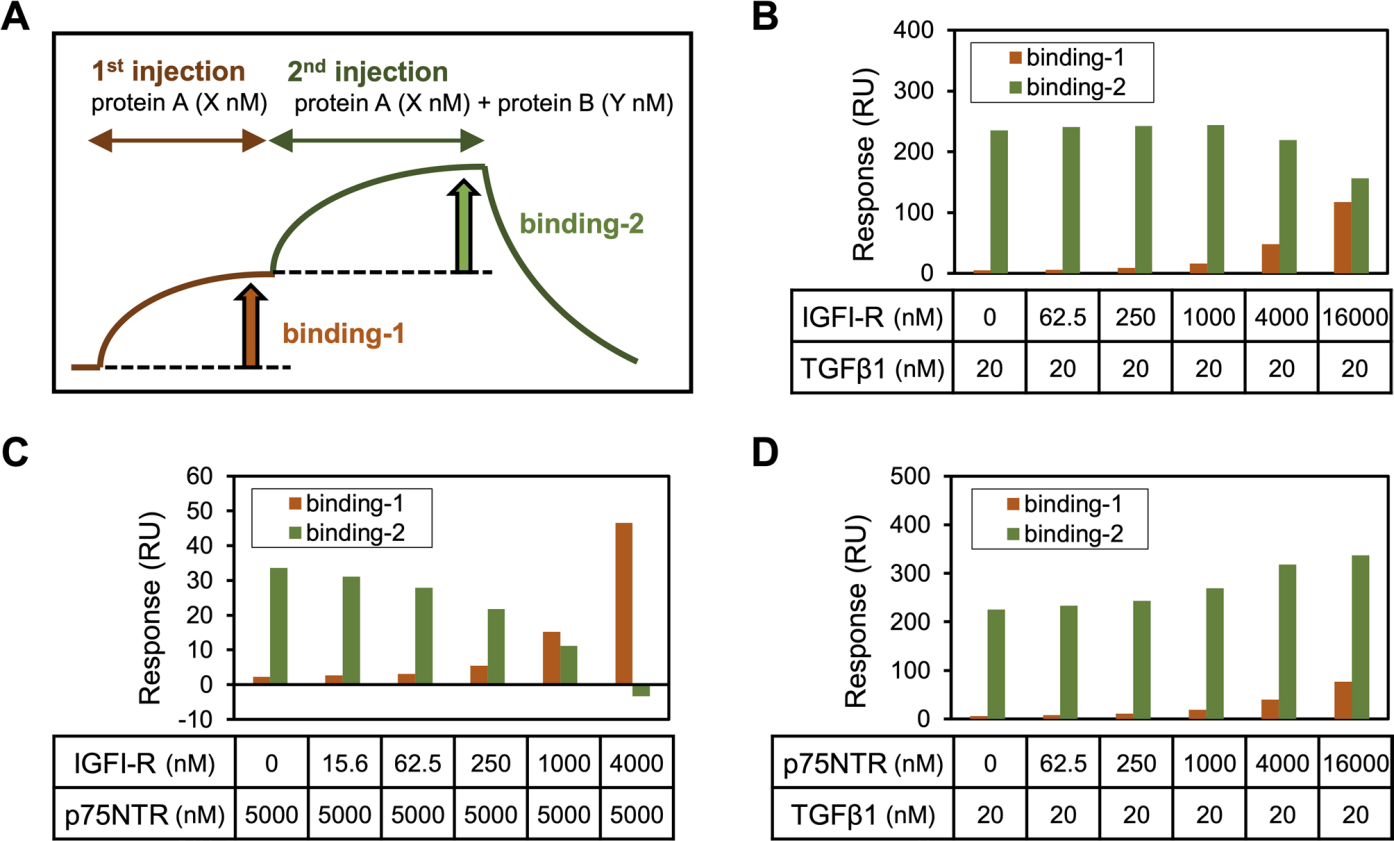
Dual injection assay. The assay was performed according to the experimental scheme shown in (**A**) using the following pairs: (**B**) IGFI-R (protein A) and TGFβ1 (protein B), (**C**) IGFI-R (protein A) and p75NTR (protein B), and (**D**) p75NTR (protein A) and TGFβ1 (protein B). These proteins were injected onto immobilized PRELP, and the binding response was measured. Orange and green bars indicate binding-1 and binding-2, respectively.

To investigate whether TGFβ1, IGFI-R, and p75NTR can bind to PRELP simultaneously, we performed a dual injection assay using SPR (Figs. 3A–D, Supplementary Fig. S7). The assay was conducted as depicted in Fig. 3A. X nM (variable) of “protein A” was first injected into PRELP immobilized on a sensor chip (binding-1), and then a mix of X nM of “protein A” with Y nM (constant) of “protein B” was injected (binding-2). We evaluated the binding-2 responses depending on the concentration change of “protein A.” Results of this assay indicated that TGFβ1 and IGFI-R competitively bind to PRELP such that the binding region of PRELP for the interaction with TGFβ1 overlaps that with IGFI-R (Fig. 3B). Similarly, IGFI-R and p75NTR competitively bound to PRELP (Fig. 3C). However, p75NTR did not inhibit but rather somewhat enhanced the binding response between TGFβ1 and PRELP (Fig. 3D). These results suggest that PRELP likely uses different but partially overlapping regions in the LRR1-7 domain for the interaction with TGFβ1, IGFI-R, and p75NTR, leading to the wide range of binding specificities of PRELP.

### Up-regulation of gene expression level of ECM proteins by PRELP

**Fig. 4:**
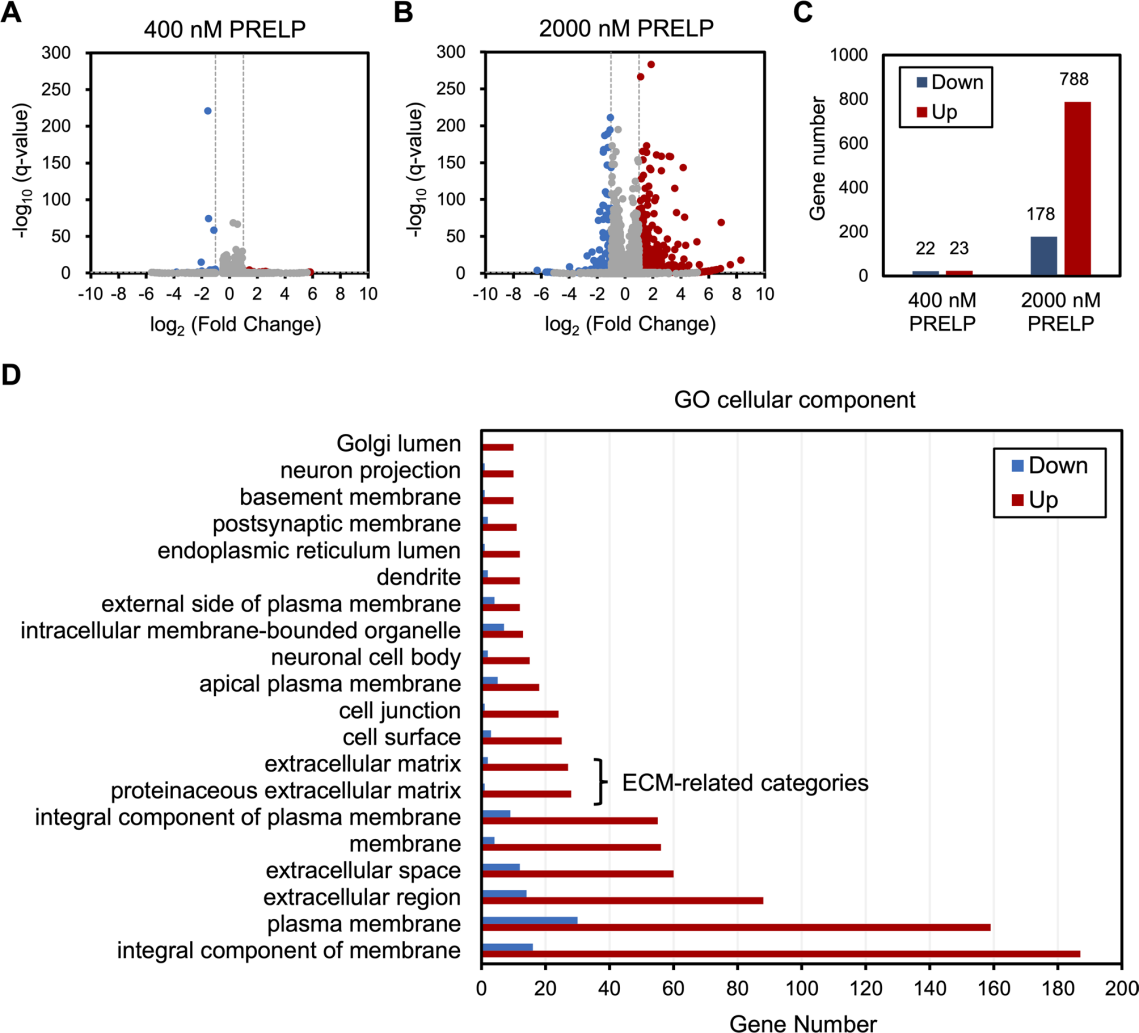
Alteration of gene expression levels by addition of recombinant PRELP. (**A**–**B**) Volcano plots from gene differential expression analysis of A549 cells treated with (**A**) 400 nM and (**B**) 2000 nM of recombinant PRELP compared with control cells. The criteria are fold change: greater than 2; q-value: less than 0.05. Significantly up- and down-regulated genes are represented as red and blue plots, respectively. (**C**) Gene numbers that are significantly up- or down-regulated. (**D**) GO cellular component analysis. Numbers of differentially expressed genes between control and 2000 nM PRELP are shown (the top 20 of up-regulated GO categories).

We previously showed that PRELP exerts physiological activities, including cell growth suppression and morphological change of A549 lung carcinoma cells, at micromolar concentrations^17^. To further assess cellular responses caused by PRELP, we quantitatively evaluated the alteration in gene expression of A549 cells treated with the recombinant PRELP in the concentration-dependent manner. RNA-seq analysis showed that 2000 nM of recombinant PRELP significantly up- or down-regulated expression levels of various genes, whereas 400 nM of the recombinant PRELP affected only a small number of gene expression levels (Figs. 4A–C, Supplementary Tables S2, S3, S4). Gene Ontology (GO) enrichment analysis indicated that 2000 nM of the recombinant PRELP up-regulated the gene expression levels of many proteins assigned to ECM-related categories (Fig. 4D, Supplementary Figs. S8, S9, Supplementary Table S5) including various ECM components, suggesting that PRELP contributes to the ECM organization.

### Localization of PRELP in the ECM

Our in vitro interaction analysis revealed the micromolar range of binding affinities for the interactions of PRELP with TGFβ1, IGFI-R, and p75NTR. RNA-seq analysis also showed that PRELP altered various gene expression levels, at micromolar concentrations. Although these investigations indicated that PRELP regulates cellular functions at the micromolar range of concentrations triggered by micromolar affinity interactions, it has not been clear whether the in vivo concentration of PRELP reaches the micromolar range. Considering that many SLRPs are localized in the ECM, we hypothesized that PRELP achieves local high concentrations in the proximity of cell surface environments by anchoring to the ECM proteins.

**Fig. 5:**
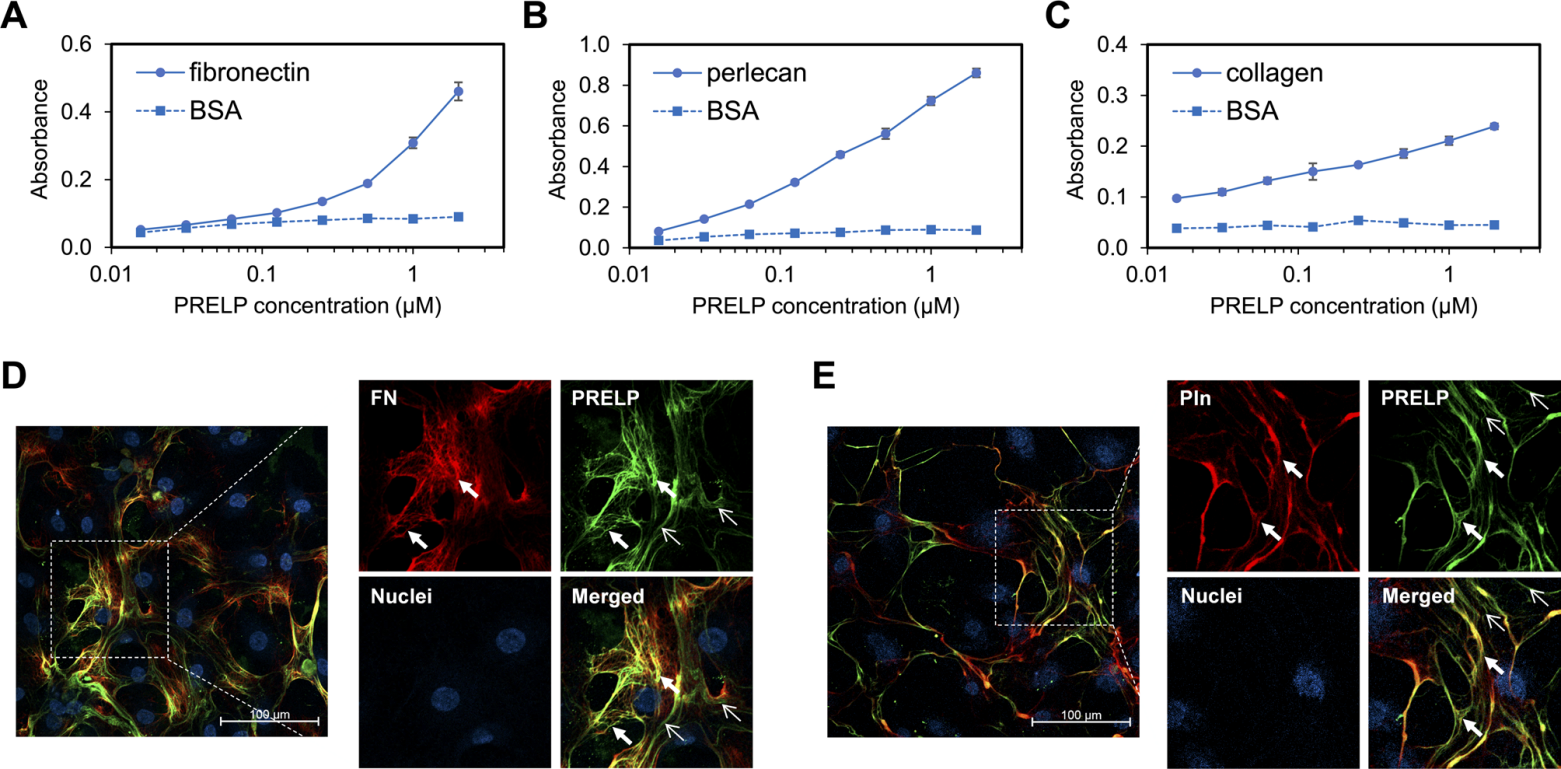
Localization of PRELP in the ECM. (**A**–**C**) ELISA direct binding analysis of PRELP with immobilized (**A**) human fibronectin, (**B**) mouse perlecan, and (**C**) bovine collagen. The binding of PRELP to BSA was measured as a negative control. (**D**–**E**) Immunofluorescence staining. HUVECs treated with 400 nM of recombinant PRELP protein were double-immunostained with (**D**) anti-PRELP antibody and anti-fibronectin antibody (FN) and (**E**) anti-PRELP antibody and anti-perlecan antibody (Pln). Nuclei were visualized by DAPI staining. Bold arrows indicate co-localization, and fine arrows indicate only or mainly PRELP localization. Scale bar: 100 μm.

To address this hypothesis, we firstly verified the interactions of PRELP with three ECM components, fibronectin, perlecan, and collagen, using ELISA. The results showed that PRELP directly bound to these ECM proteins in the concentration-dependent manner (Figs. 5A–C). We further assessed co-localization of PRELP with the ECM components through immunofluorescence staining using human umbilical vein endothelial cells (HUVECs) treated with recombinant PRELP proteins. The double-immunofluorescence staining of PRELP and fibronectin or perlecan showed co-localization of PRELP with these ECM components and, therefore, PRELP incorporation into the ECM (Figs. 5D–E, Supplementary Figs. S10, S11), although collagen I was not detected due to the low expression level in HUVECs (Supplementary Fig. S12). These results indicate that PRELP proteins are accumulated in the ECM through the interactions with several ECM components.

## Discussion

In this study, we validated the multi-specific direct binding of PRELP to TGFβ1, IGFI-R, and p75NTR with relatively weak affinities around the micromolar range using SPR analysis (Figs. 1A–C). Our competitive binding analysis also indicated that PRELP competitively interacted with TGFβ1 against the binding between TGFβ1 and TGFβRII (Fig. 1F) in agreement with the function of PRELP as an inhibitor of TGFβ signaling as reported previously^13,16^. Although comparison of kinetic parameters for the interaction with TGFβ1 showed that PRELP had faster on-rate and off-rate than TGFβRII, the binding affinities were comparable (Table 1). This result illustrates that PRELP can moderately regulate TGFβ1 signaling depending on the local concentration of each protein. It should be also noted that our in vitro interaction analysis did not show direct binding between PRELP and the TGFβRII ectodomain in contradiction to a previous study^16^, although the TGFβ1-TGFβRII interaction was observed. This difference was possibly due to different experimental conditions and methods.

We also investigated the binding regions of PRELP that are involved in the interactions with multiple ligands using N-terminal-truncated and chimeric PRELP constructs and indicated that PRELP bound to TGFβ1, IGFI-R, and p75NTR through the LRR1-7 region (Fig. 2). As for the interaction parameters, the binding affinity of TGFβ1 for ΔNT50 was a little weaker than that for full-length PRELP, and similarly chPRELP showed slightly weaker affinities for the interactions with TGFβ1 and IGFI-R compared with full-length PRELP (Tables 2 and 3), suggesting that the deficient regions also contribute to the interactions in addition to the LRR1-7 region. Our dual injection assay indicated that the interaction of PRELP with IGFI-R competed with the binding to TGFβ1 and p75NTR (Figs. 3B–C). In contrast, p75NTR binding did not compete with the interaction between PRELP and TGFβ1 and instead increased the binding response of TGFβ1 (Fig. 3D). Although SPR analysis showed that the binding affinity between p75NTR and TGFβ1 was weak (Supplementary Fig. S13), this interaction might be stabilized by formation of the ternary complex with PRELP. Collectively, these results suggest that PRELP simultaneously binds to the secreted protein TGFβ1 and the membrane receptor p75NTR, which might lead to non-canonical signaling transduction. Our results suggest that the binding sites in the LRR1-7 region of PRELP that are involved in the interactions with TGFβ1, IGFI-R, and p75NTR differ but partially overlap so that PRELP achieves a wide range of specificity for multiple target proteins through different binding mechanisms. Because TGFβ1 is a common ligand of many SLRPs^1–3^, it likely recognizes some highly conserved amino acids in the LRR domain of SLRP family. Although details of the molecular mechanisms of the PRELP interactions remain to be validated, the concave surface and the adjacent loops are most commonly responsible for the ligand interactions in many LRR proteins^21^ and might be also important for the interactions of PRELP. Further characterization of the interactions of PRELP with a variety of ligands will lead to a better understanding of context-dependent functional networks of PRELP.

RNA-seq analysis indicated that PRELP significantly up-regulated the gene expression levels of various ECM components including fibronectin and collagen in A549 cells at the micromolar concentration (Fig. 4, Supplementary Tables S4, S5), suggesting that these alteration of gene expressions were emanated from the weak interactions with receptors. To support our hypothesis that PRELP contributes to ECM organization, a previous study showed the disruption of ECM structure in the retina of *PRELP*^−/−^ knockout mice^10^. In addition to ECM components, PRELP enhanced the expression of various matrix metalloproteinases (Supplementary Table S4), which disassemble ECM components, suggesting that PRELP may modulate the ECM composition to make it favorable for PRELP. PRELP also up-regulated the expression level of many integrins (Supplementary Table S4), which are known as ECM receptors and govern the cell-matrix interactions, in agreement with a previous study that indicates positive correlation between PRELP and integrin expression^12^. RNA-seq analysis also showed great up-regulation of the gene expression levels of many proteins related to cell adhesion (Supplementary Fig. S8), suggesting that PRELP exerts the tumor suppressive function through the regulation of cell adhesion as indicated previously^10,13^.

**Fig. 6:**
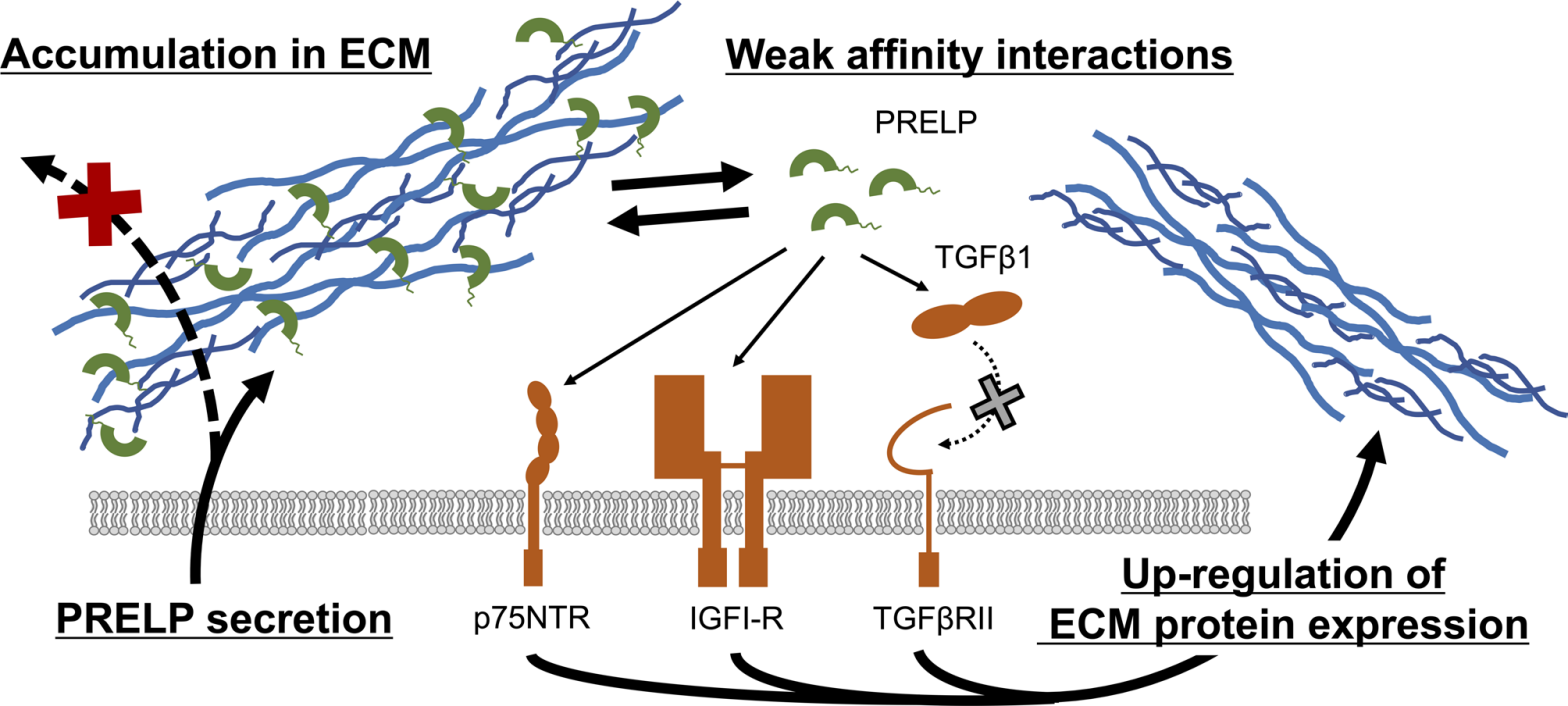
Proposed model of low affinity interactions of PRELP achieved by local protein accumulation through ECM anchoring and enhancement of ECM organization by PRELP.

ELISA binding analysis and immunofluorescence staining showed that PRELP directly interacted with fibronectin, perlecan, and collagen (Figs. 5A–C), and that it localized to the ECM in HUVECs treated with the recombinant PRELP protein (Figs. 5D–E). The results of ELISA indicated that PRELP more strongly bound to perlecan than to fibronectin, and in agreement with this result, PRELP was more clearly co-localized with perlecan than with fibronectin. Binding strength between immobilized perlecan and soluble PRELP in this study was likely weaker than the affinity between immobilized PRELP and soluble perlecan measured by SPR analysis in a previous study^8^. Since PRELP interacts with heparan sulfate chains on perlecan, and the heparan sulfate chains contain a number of disaccharide repeats leading to the avidity effects, the calculated *K*_D_ values of the interaction of soluble perlecan in the previous study would be apparently small. Although endogenous PRELP was not observed in control cells without PRELP supplementation (Supplementary Figs. S10, S11), this result was probably due to the low expression level of endogenous PRELP protein in HUVECs, as indicated by The Human Protein Atlas^22^. Consistent with our data, previous studies demonstrated that PRELP has a wide distribution among various connective tissues, which are rich in ECM^23,24^. Previous confocal immunohistochemistry studies also showed that PRELP was located adjacent to basement membranes in connective tissues^8^. Our finding suggests that the anchoring of PRELP to ECM components increases the apparent local concentration pericellularly so that PRELP interactions can occur in spite of intrinsic weak micromolar binding affinities (Fig. 6). Without the ECM anchoring, secreted PRELP proteins would not accumulate but diffuse out of the cells. In ELISA analysis, the binding response of PRELP to the ECM proteins increased monotonically in the concentration range from 10^− 8^ M to 10^− 6^ M and did not reach saturation even at a micromolar concentration, indicating that PRELP does not interact so strongly with ECM components. Given that a previous study also showed that a member of class II SLRPs osteomodulin, which has 44% sequence homology with PRELP, binds to type I collagen by a weak binding affinity due to the fast association and dissociation^4^, PRELP might be also in equilibrium between ECM-bound and unbound states with the moderate affinities and kinetics so that the ECM-unbound PRELP can interact with target ligands. Alternatively, it is possible that PRELP interacts with both ECM proteins and the target ligands simultaneously. Considering that PRELP binds to collagen through the LRR domain and to perlecan through the N-terminal region as indicated previously^8^, it is expected that ECM-bound PRELP interacts with some target ligands depending on which ECM proteins PRELP is anchored to. Furthermore, RNA-seq analysis showed that PRELP up-regulated the gene expression levels of various ECM components, suggesting that PRELP enhances the ECM organization to further facilitate the weak affinity interactions of PRELP. Intriguingly, it is known that ECM degradation and remodeling occur to create a favorable microenvironment for the progression of tumorigenesis and metastasis^25,26^. During the ECM degradation process, local concentrations of PRELP that were anchored to the ECM would be reduced, which might result in disruption of low affinity interactions and tumor suppressive functions of PRELP. It has been recently considered that weak and multivalent interactions play critical roles in living cells through the protein compartmentalization including the liquid‒liquid phase separation, which elevates protein concentrations at defined moments and spaces^27,28^, and our finding suggests that ECM also provides an interaction platform in which ECM-localized proteins can exert the functions triggered by the weak binding affinities.

In conclusion, our results suggest that PRELP is likely to have diverse molecular functions triggered by micromolar affinity interactions with multiple targets, including TGFβ1, IGFI-R, and p75NTR. For physiological activities, PRELP protein concentrations are likely to be locally enhanced through ECM localization and thereby accomplish the appropriate functions. PRELP also likely promotes the ECM organization to maintain the preferred environment to exert the molecular functions. Such a complex mechanism, namely the combination of relatively weak affinity to each ligand and anchoring-dependent modulation of the interactions, likely enable PRELP to contribute to the fine-tuning of biological homeostasis in a context-dependent manner. The findings of this study are beneficial for understanding of the molecular functions of PRELP through weak affinity interactions.

## Methods

### Expression and purification of Recombinant PRELP proteins

Recombinant human full-length PRELP was prepared as described previously^17^. Briefly, human full-length PRELP (amino acid residues 21–382 corresponding to the numbering in the Uniprot database^29^) with a FLAG-tag at the C-terminus and a sp1–2 signal sequence^30^ at the N-terminus was produced using Mimic Sf9 cells (Thermo Fisher Scientific, Waltham, MA, USA). The recombinant PRELP was purified from the supernatant using DDDDK-tagged Protein Purification Gel (MBL, Tokyo, Japan) followed by treatment with Benzonase nuclease (Millipore, Burlington, MA, USA) overnight to remove the contamination of nucleic acids. After a second purification using DDDDK-tagged Protein Purification Gel, the eluate was purified by size-exclusion chromatography. We also expressed and purified N-terminal-truncated PRELP ΔNT24 and ΔNT50, which lack the N-terminal 24 and 50 amino acid residues, respectively, following the same procedure, except that Benzonase treatment was not performed because nucleic acid contamination was not observed in the ΔNT24 and ΔNT50 samples.

Recombinant chimeric PRELP, which is composed of a portion of human PRELP (amino acid residues 1–268, Uniprot) and a portion of listeria monocytogenes internalin B (amino acid residues 189–248, Uniprot), with a FLAG-tag at the C-terminus was produced using Expi293 cells (Thermo Fisher Scientific). The DNA sequence was subcloned into the pcDNA3.4 vector (Thermo Fisher Scientific), and Expi293 cells were transfected with the expression vector using the ExpiFectamine 293 Transfection Kit (Thermo Fisher Scientific) in accordance with the manufacturer’s protocol. The cells were cultured for 3 days at 37 °C and 8% CO_2_. From the culture supernatant, the recombinant chimeric PRELP was purified by FLAG-tag purification and subsequent size exclusion chromatography, following the same procedure as the purification of N-terminal-truncated PRELP.

### Expression and purification of Recombinant TGFβ1

The DNA sequence encoding human TGFβ1 pro-protein (amino acid residues 30–390, Uniprot) with an octahistidine tag at the N-terminus was subcloned into the pcDNA3.4 vector (Thermo Fisher Scientific). To increase the expression level of TGFβ1, we used the signal sequence of rat serum albumin as the signal peptide, and a cysteine residue at position 33 of the TGFβ1 pro-protein was replaced with a serine residue as reported previously^31^. ExpiCHO cells were transfected with the expression vector using the ExpiFectamine CHO Transfection Kit (Thermo Fisher Scientific) in accordance with the manufacturer’s max-titer protocol. The cells were cultured for 2 weeks at 32°C and 5% CO_2_. The culture was centrifuged at 1000 × g for 10 min, and the supernatant was collected and filtered. The recombinant mature TGFβ1 was subsequently purified following a previously described methodology^32^ with some modifications. The collected supernatant was applied to a Ni-NTA agarose affinity column (Qiagen, Hilden, Germany) equilibrated with binding buffer (50 mM Tris-HCl (pH 8.0), 150 mM NaCl, 5 mM imidazole). The column was first washed with the binding buffer, and fractions containing latent TGFβ1 were eluted with buffers containing increasing concentrations of imidazole (20–500 mM). For activation, the eluate was dialyzed against 100 mM acetic acid after addition of appropriate amount of 6 M HCl to adjust the eluate pH to below 3.0. The recombinant mature TGFβ1 dimer was finally isolated by cation exchange chromatography using a RESOURCE S column (Cytiva, Marlborough, MA, USA). Mobile phase A was 20 mM sodium acetate containing 30% (v/v) 2-propanol at pH 4.0, and mobile phase B was 20 mM sodium acetate, 500 mM NaCl containing 30% (v/v) 2-propanol at pH 4.0. A linear gradient was used (0 to 95% B).

### Expression and purification of the Recombinant TGFβRII extracellular domain

The extracellular domain of TGFβRII was produced by *Escherichia coli* and purified in accordance with refolding methods reported previously^32,33^ with some modifications. The DNA sequence encoding the extracellular domain of human TGFβRII (amino acid residues 38–159, Uniprot) with a hexahistidine tag at the N-terminus was subcloned into the pET28b vector. *E. coli* strain Rosetta2 (DE3) transformed with the vector was grown overnight at 28 °C in 100 mL of 2×YT medium. The cells were further grown in 1 L of 2×YT medium and cultured at 28 °C. When the OD_600_ reached 0.6–0.8, isopropyl β-D-1-thiogalactopyranoside was added so that the final concentration was 0.8 mM. After the cells were cultured with shaking at 100 rpm overnight at 20 °C, the cells were harvested by centrifugation at 7000 × g for 10 min at 4 °C. The collected cells were resuspended in disruption buffer (100 mM Tris-HCl (pH 8.0) containing 1 mM phenylmethylsulfonyl fluoride) and lysed using an ultrasonic cell-disrupting UD-201 instrument (TOMY, Tokyo, Japan). The cell lysate was centrifuged at 20000 × g for 20 min at 4 °C. The insoluble pellet was resuspended in the disruption buffer containing 1 M NaCl and centrifuged at 20000 × g for 20 min at 4 °C. The pellet was resuspended in the disruption buffer containing 2% (v/v) Triton X-100 and rotated for 30 min at 4 °C. After centrifugation at 7300 × g for 30 min at 4 °C, the pellet was washed by two cycles of resuspension in water and centrifugation at 7300 × g for 30 min at 4 °C. The pellet was collected as the final inclusion bodies.

The inclusion bodies were dissolved in solubilizing buffer (6 M guanidine-HCl, 20 mM Tris-HCl (pH 8.0), 1 mM dithiothreitol) and rotated overnight at 4 °C. The solubilized sample was centrifuged at 10000 × g for 30 min at 4 °C, and the supernatant was collected and applied to a Ni-NTA agarose affinity column (Qiagen) equilibrated with the solubilizing buffer. The column was first washed with the solubilizing buffer containing 5 mM imidazole, and proteins were eluted with the buffers containing increasing concentrations of imidazole (20–500 mM). The eluate was mixed with 10 mM dithiothreitol and incubated at room temperature for 4 h shielded from light. The sample was concentrated, slowly injected into refolding buffer (100 mM Tris-HCl (pH 8.0), 500 mM arginine-HCl, 2 mM reduced-glutathione, 0.5 mM oxidized-glutathione) (100-fold dilution), and gently stirred for 16–18 h at 4 °C. The solution was dialyzed against 10 mM Tris-HCl (pH 8.0) (20-fold dialysis, twice). The solution was applied to a Ni-NTA agarose affinity column (Qiagen) equilibrated with binding buffer (50 mM Tris-HCl (pH 8.0), 50 mM NaCl, 5 mM imidazole). The column was first washed with the binding buffer containing 5 mM imidazole, and proteins were eluted with the buffers containing increasing concentrations of imidazole (20–500 mM). The eluate was finally purified by size-exclusion chromatography using a HiLoad 16/600 Superdex 75 pg column (Cytiva) equilibrated with 50 mM Tris-HCl (pH 8.0), 50 mM NaCl.

### Expression and purification of the Recombinant IGFI-R and p75NTR extracellular domain

Recombinant proteins of the extracellular domain of human IGFI-R (amino acid residues 31–935, Uniprot) and human p75NTR (amino acid residues 29–189, Uniprot) with a hexahistidine tag at the C-terminus were prepared as described previously^17^. Briefly, recombinant IGFI-R and p75NTR were produced using ExpiCHO cells and Sf9 cells (Thermo Fisher Scientific), respectively. From the culture supernatant, the proteins were purified by Ni-NTA agarose affinity column and subsequent size-exclusion chromatography.

### SPR

The direct binding assay using SPR was conducted using a Biacore T200 instrument (Cytiva). Recombinant PRELP proteins and TGFβ1 were immobilized using an amine coupling method as follows. A CM5 Biacore sensor chip (Cytiva) was activated by treatment with N-hydroxysuccinimide/N-ethyl-N´-(3-dimethylaminopropyl) carbodiimide hydrochloride, followed by immobilization at around 400 (full-length PRELP, ΔNT24, and ΔNT50 for the interaction with TGFβ1 and TGFβRII), 4000 (full-length PRELP, ΔNT24, and ΔNT50 for the interaction with IGFI-R and p75NTR), 600 (chPRELP for the interaction with TGFβ1 and IGFI-R), 2000 (chPRELP for the interaction with p75NTR), or 200 (TGFβ1) resonance units (RU). After the immobilization, the activated surface of the sensor chip was blocked with 1 M ethanolamine hydrochloride (pH 8.5). Recombinant TGFβ1, TGFβRII, IGFI-R, or p75NTR was injected into the sensor chip at a flow rate of 30 µL/min; a range of concentrations was tested. The protein concentrations were calculated from the absorbance at 280 nm divided by the extinction coefficients based on the amino acid sequences. The association time was 90 s, and the dissociation time was 180 s (TGFβ1 and TGFβRII) or 240 s (IGFI-R and p75NTR). The assay was carried out in 10 mM HEPES (pH 7.5), 150 mM NaCl, containing 0.05% (v/v) Tween-20 at 15 °C. To reduce non-specific binding, 50 mM arginine-HCl was also added to the buffer for the assay using TGFβ1 and TGFβRII. A regeneration procedure was performed at the end of each cycle with 1 M arginine-HCl (pH 4.4). The data were analyzed using BIAevaluation software (Cytiva). Kinetic parameters and dissociation constants were obtained by global fitting or steady-state equilibrium analysis. The SPR measurements between PRELP and TGFβ1 were also collected at a range of temperatures (10–22 °C), and the changes in enthalpy (Δ*H°*) and entropy (Δ*S°*) were calculated as described previously^34^.

### Competitive binding analysis

Competitive binding analysis was carried out using SPR. Recombinant full-length PRELP was immobilized on a CM5 sensor chip at around 4000 RU using the amine coupling method. A mix of 1 µM of TGFβ1 with a range of concentrations of TGFβRII was injected into the sensor chip at a flow rate of 30 µL/min. The association time was 90 s, and the dissociation time was 180 s. The assay was carried out in 10 mM HEPES (pH 7.5), 150 mM NaCl, 50 mM arginine-HCl containing 0.05% (v/v) Tween-20 at 15 °C. A regeneration procedure was performed at the end of each cycle with 1 M arginine-HCl (pH 4.4). The binding response was evaluated using BIAevaluation software (Cytiva).

### Dual injection assay

The dual injection assay was performed using SPR referring to previously described methods^35–37^ and the dual injection method of the Biacore system. First, a range of concentrations (X nM) of “protein A” was injected into recombinant full-length PRELP immobilized on a CM5 sensor chip at around 4000 RU at a flow rate of 30 µL/min for 60–90 s until the binding response reached equilibrium (binding-1). Next, a mix of the same range of concentrations (X nM) of “protein A” with a fixed concentration (Y nM) of “protein B” was injected for 90 s (binding-2). The dissociation time was 240 s after the binding-2 process. The dual injection assay was carried out in 10 mM HEPES (pH 7.5), 150 mM NaCl containing 0.05% (v/v) Tween-20 at 15 °C. A regeneration procedure was performed at the end of each cycle with 1 M arginine-HCl (pH 4.4). The following pairs were analyzed: (i) IGFI-R (protein A) and p75NTR (protein B); (ii) IGFI-R (protein A) and TGFβ1 (protein B); and (iii) p75NTR (protein A) and TGFβ1 (protein B). The responses of binding-1 and binding-2 were analyzed using BIAevaluation software (Cytiva).

### CD

To analyze the secondary structure of recombinant full-length PRELP, ΔNT24, ΔNT50, and chPRELP, CD spectra were measured by a J-820 spectropolarimeter (Jasco, Oklahoma City, OK, USA) using the sample prepared at 2 µM in 10 mM HEPES pH 7.5, 150 mM NaCl at 20 °C. CD measurements were performed at a scanning speed of 20 nm/min by continuous scanning from 260 to 200 nm using a 1 mm path-length quartz cell. Five scans were accumulated, and the collected data were analyzed using Spectra Analysis software (Jasco) and smoothed using a Savitzky-Golay method.

### RNA-seq

Suspension of A549 cells (RIKEN) were seeded in 10 cm dishes for 2 × 10^5^ cells/dish and pre-incubated in DMEM containing 10% (v/v) fetal bovine serum for 24 h at 37 °C and 5% CO_2_. Following the preincubation, the medium was replaced with new medium containing different concentrations (0, 400, or 2000 nM) of recombinant full-length PRELP. Independent two cell cultures were prepared for each concentration, and the cells were cultured for 48 h at 37 °C and 5% CO_2_. The culture supernatant was removed, and the cells were lysed with TRIzol Reagent (Thermo Fisher Scientific) after washing with phosphate buffered saline (PBS) at pH 7.4.

Transcriptome sequencing experiments including RNA extraction and quality control, library construction, purification, library quality control and quantitation, sequencing cluster generation, and high through-put sequencing were processed and analyzed by GENEWIZ from Azenta Life Sciences using Illumina platform. In data analysis, to remove technical sequences, including adapters, PCR primers, or fragments thereof, and quality of bases lower than 20, pass filter data of FASTQ format were processed by Cutadapt (version 1.9.1) to be high quality clean data. For mapping, reference genome sequences (Homo sapiens) and gene model annotation files of relative species were firstly downloaded from genome website, such as UCSC, NCBI, ENSEMBL, and secondly, Hisat2 (version 2.0.1) was used to index reference genome sequence. Finally, clean data were aligned to reference genome via software Hisat2 (version 2.0.1). For expression analysis, transcripts in FASTA format are converted from known GTF annotation file and indexed properly, and then, with the file as a reference gene file, HTSeq (version 0.6.1) estimated gene and isoform expression levels from the pair-end clean data. Differential expression analysis used the DESeq2 Bioconductor package, a model based on the negative binomial distribution. The estimates of dispersion and logarithmic fold changes incorporate data-driven prior distributions, and Padj of genes were set < 0.05 to detect differential expressed ones. The results were further analyzed to determine genes with significant differential expression according to the criteria of fold change greater than 2 and q-value less than 0.05. GOSeq (version 1.34.1) was used identifying Gene Ontology terms that annotate a list of enriched genes with a significant Padj less than 0.05.

### ELISA

Microtiter wells (Sumitomo Bakelite, Tokyo, Japan, MS-8496 F) were coated with 80 µL of human fibronectin (Sigma Aldrich, St. Louis, MO, USA, F0895) at 5 µg/mL in PBS, mouse perlecan (Sigma Aldrich, H4777) at 1 µg/mL in PBS, and bovine collagen (KOKEN, Tokyo, Japan, AteloCell I-AC) at 5 µg/mL in PBS overnight at 4 °C. The wells were washed three times with washing buffer (10 mM HEPES pH 7.5, 150 mM NaCl, 0.05% (v/v) Tween-20) and blocked with the washing buffer containing 10% (w/v) bovine serum albumin (BSA) at room temperature for 2 h. The wells were washed three times with the washing buffer and then incubated with a range of concentrations of recombinant full-length PRELP in the washing buffer at room temperature for 1 h. After washing three times with the washing buffer, bound PRELP was detected with anti-DDDDK-tag mAb HRP-DirecT (MBL, M185-7) (1:1000 in the washing buffer containing 5% (w/v) BSA) at room temperature for 1 h. The wells were washed three times with the washing buffer and developed with TMB soluble Reagent (ScyTek Laboratories, Logan, UT, USA). The reaction was stopped by adding TMB Stop Buffer (ScyTek Laboratories) to the wells, and the plates were read at 450 nm using an ARVO-X3 instrument (PerkinElmer, Waltham, MA, USA). For each experiment, the binding of PRELP to immobilized BSA was also measured as a negative control.

### Immunofluorescence staining

HUVECs (Lonza, Basel, Switzerland) were used for immunofluorescence staining. A suspension of 2 × 10^4^ cells/well was seeded in 8-well chamber slides (Watson, San Diego, CA, USA) and cultured in EGM-2 medium (Lonza) for a week at 37 °C and 5% CO_2_. The medium was replaced with fresh medium every 3 days. Next, 0, 400, or 2000 nM of recombinant full-length PRELP was added to the wells 2 days before cell fixation. The culture supernatant was removed, and the cells were fixed by incubation with PBS (pH 7.4) containing 4% (v/v) paraformaldehyde for 10 min at room temperature. After the removal of solutions, the wells were washed three times with PBS (pH 7.4) for 5 min. The wells were blocked with PBS (pH 7.4) containing 5% (w/v) BSA for 30 min at room temperature. After removal of the solutions and washing with PBS (pH 7.4) for 5 min, the cells were double-immunostained with mouse anti-PRELP antibody (obtained in our laboratory, 10 µg/mL) and rabbit anti-fibronectin antibody (Abcam, ab2413, 1:200, Cambridge, UK), rat anti-perlecan antibody (Santa Cruz Biotechnology, sc-33707, 1:200, Dallas, TX, USA), or rabbit anti-collagen I antibody (Cell Signaling Technology, #39952, 1:200, Danvers, MA, USA) diluted in PBS (pH 7.4) containing 2% (w/v) BSA at 4 ^o^C overnight. After removal of the solutions, the wells were washed twice with PBS (pH 7.4) for 5 min and once with PBS (pH 7.4) containing 0.01% (v/v) Tween-20 (PBS-T) for 5 min. They then were incubated with PBS-T containing anti-mouse IgG Alexa Fluor 488 (Thermo Fisher Scientific, A11001, 1:200) and anti-rabbit IgG Alexa Fluor 594 (Thermo Fisher Scientific, A21207, 1:200) or anti-rat IgG Alexa Fluor 594 (Thermo Fisher Scientific, A21209, 1:200) for 1 h at room temperature shielded from light. The wells were washed three times with PBS (pH 7.4) for 5 min, and slides were mounted using ProLong Gold Antifade Mountant with DNA Stain DAPI (Thermo Fisher Scientific). The immunofluorescence images were obtained using a LSM 800 Airyscan confocal microscope and analyzed using ZEN blue software (both from ZEISS, Oberkochen, Germany).

## Electronic supplementary material

Below is the link to the electronic supplementary material.

Supplementary Material 1

Supplementary Material 2

Supplementary Material 3

Supplementary Material 4

Supplementary Material 5

## Data Availability

The RNA-seq raw datasets analyzed during the current study are available in the Mendeley Data repository, with the data set by following link; https://doi.org/10.17632/38pcrz36rd.1 and https://doi.org/10.17632/cfykmyzv82.1. The other datasets used and/or analyzed during the current study are available from the corresponding author on reasonable request.

## Abbreviations

BSA: bovine serum albumin
CD: circular dichroism
ECM: extracellular matrix
HUVECs: human umbilical vein endothelial cells
IGFI-R: insulin-like growth factor I receptor
LRR: leucine-rich repeat
mAb: monoclonal antibody
p75NTR: low-affinity nerve growth factor receptor
PBS: phosphate buffered saline
PRELP: proline/arginine-rich end leucine-rich repeat protein
RU: resonance units
SLRPs: small leucine-rich repeat proteoglycans
SPR: surface plasmon resonance
TGFβ: transforming growth factor-beta
TGFβRII: TGF-beta receptor type-2
