# Supplementary material for: PRELP functions via multiple interactions with intrinsically weak affinity relying on ECM anchoring and remodeling
Source: Sci Rep. 2025 Jul 9;15:24634. doi: 10.1038/s41598-025-09018-7 (PMC12241546; doi:10.1038/s41598-025-09018-7)
Supplement: Supplementary file 5 — Supplementary Material 5 [file 41598_2025_9018_MOESM5_ESM.pdf]

Supplementary information for:

# PRELP Functions via Multiple Interactions with Intrinsically Weak Affinity Relying on ECM Anchoring and Remodeling

Hirofumi Kosuge[1], Makoto Nakakido[1, 2], Susana de Vega[3], Shin-ichi Ohnuma[4], Kouhei Tsumoto[1, 2, 3, *]

[1] Department of Bioengineering, School of Engineering, The University of Tokyo, 7-3-1, Hongo, Bunkyo-ku, Tokyo 113-8656, Japan

[2] Department of Chemistry and Biotechnology, School of Engineering, The University of Tokyo, 7-3-1, Hongo, Bunkyo-ku, Tokyo 113-8656, Japan

[3] The Institute of Medical Science, The University of Tokyo, 4-6-1, Shirokanedai, Minato-ku, Tokyo 108-8639, Japan

[4] The Institute of Ophthalmology, University College London, London EC1V 9EL, United Kingdom

[*] Correspondence and requests for materials should be addressed to K.T. (tsumoto@bioeng.t.u-tokyo.ac.jp)

**Table S1.** Dissociation constants ($K_D$) of the interaction of TGFβ1 with PRELP at each temperature.

| | 10 °C | 13 °C | 16 °C | 19 °C | 22 °C |
|---|---|---|---|---|---|
| $K_D$ ($\times 10^{-7}$ M) | 2.12 ± 0.07 | 2.74 ± 0.13 | 3.30 ± 0.09 | 3.98 ± 0.16 | 4.64 ± 0.07 |

Three independent measurements were carried out. The average values with standard errors are shown.

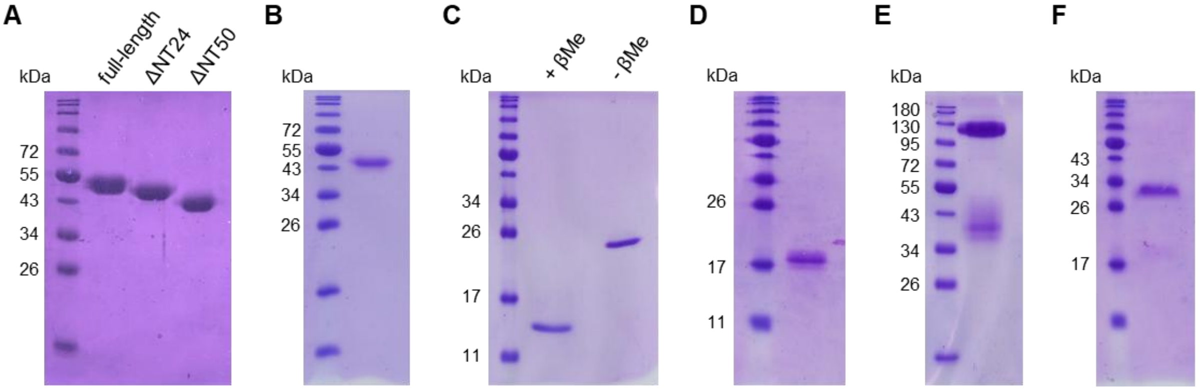


**Figure S1.** SDS-PAGE of recombinant proteins. (A) Full-length PRELP, ΔNT24, and ΔNT50 under reducing conditions. (B) chPRELP under reducing conditions. (C) TGFβ1 under reducing (+βMe) and non-reducing (-βMe) conditions. (D) TGFβRII under reducing conditions. (E) IGFI-R under reducing conditions. (F) p75NTR under reducing conditions.

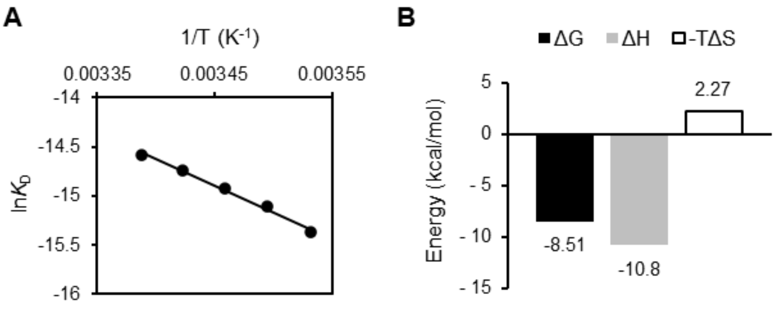


**Figure S2.** Thermodynamic parameters of the interactions between PRELP and TGFβ1 measured by SPR. (A) Van't Hoff plots of the interaction between PRELP and TGFβ1. Three independent SPR measurements at each temperature were carried out. The average values of $K_D$ at each temperature are shown. The $R^2$ value is > 0.99. (B) Thermodynamic parameters for the interaction between PRELP and TGFβ1 determined from van't Hoff plots.

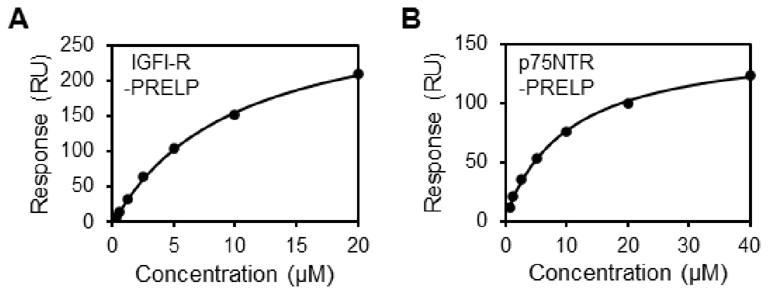


**Figure S3.** Response units vs. concentration of (A) IGFI-R and (B) p75NTR in direct binding assay with PRELP using SPR.

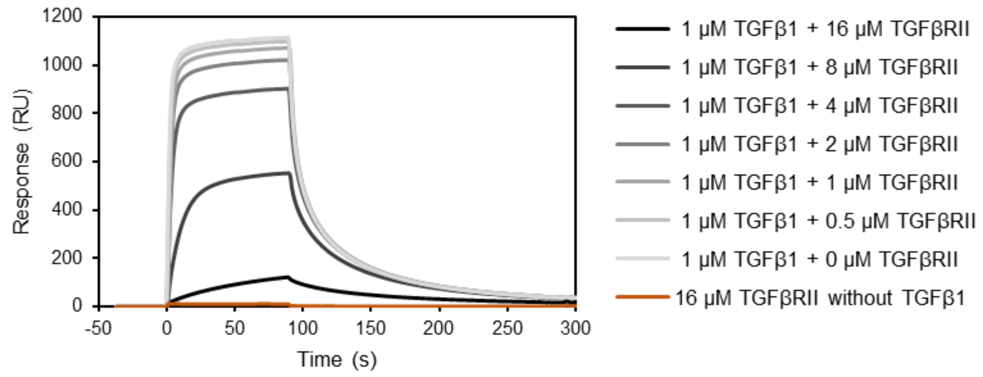


**Figure S4.** SPR raw sensorgrams of competitive binding analysis. A mix of TGFβ1 with a range of concentrations of TGFβRII was injected into PRELP immobilized on a sensor chip.

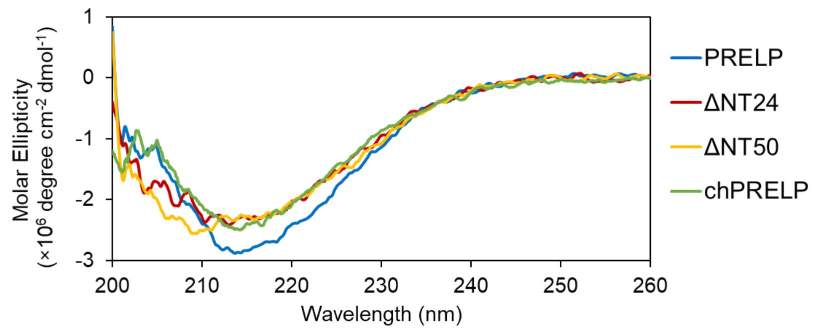


**Figure S5.** CD spectra of full-length PRELP (blue), ΔNT24 (red), ΔNT50 (yellow), and chPRELP (green).

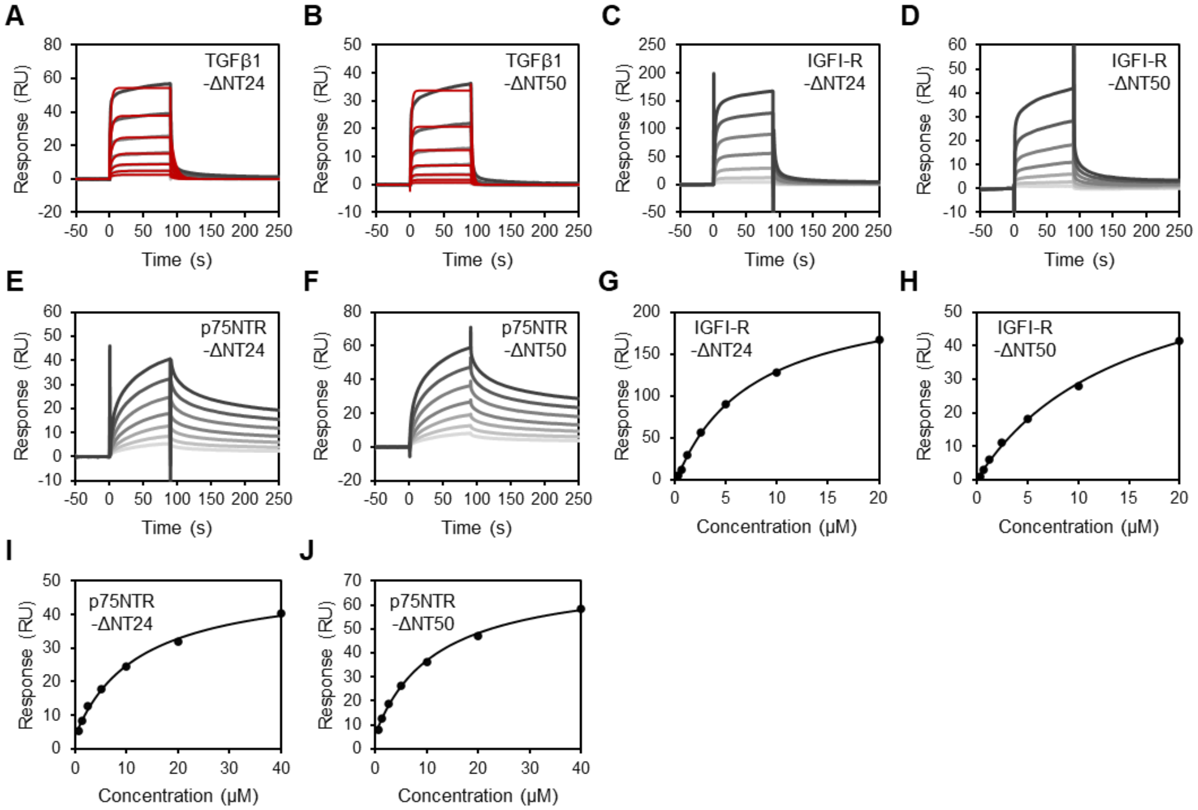


**Figure S6.** Direct binding assay of N-terminal-truncated PRELP using SPR. (A–F) SPR response vs. time curves for interactions between (A) TGFβ1 and ΔNT24, (B) TGFβ1 and ΔNT50, (C) IGFI-R and ΔNT24, (D) IGFI-R and ΔNT50, (E) p75NTR and ΔNT24, and (F) p75NTR and ΔNT50 at 15 °C. A range of concentrations of TGFβ1 (15.6–1,000 nM), IGFI-R (312.5–20,000 nM), and p75NTR (625–40,000 nM) was injected into immobilized ΔNT24 and ΔNT50. The raw sensorgrams and curve fitting profiles are shown as monochrome and red lines, respectively. Line darkness of raw sensorgrams indicates concentration. The darkest line indicates the highest concentration. (G-J) Response units vs. concentration for interactions between (G) IGFI-R and ΔNT24, (H) IGFI-R and ΔNT50, (I) p75NTR and ΔNT24, and (J) p75NTR and ΔNT50.

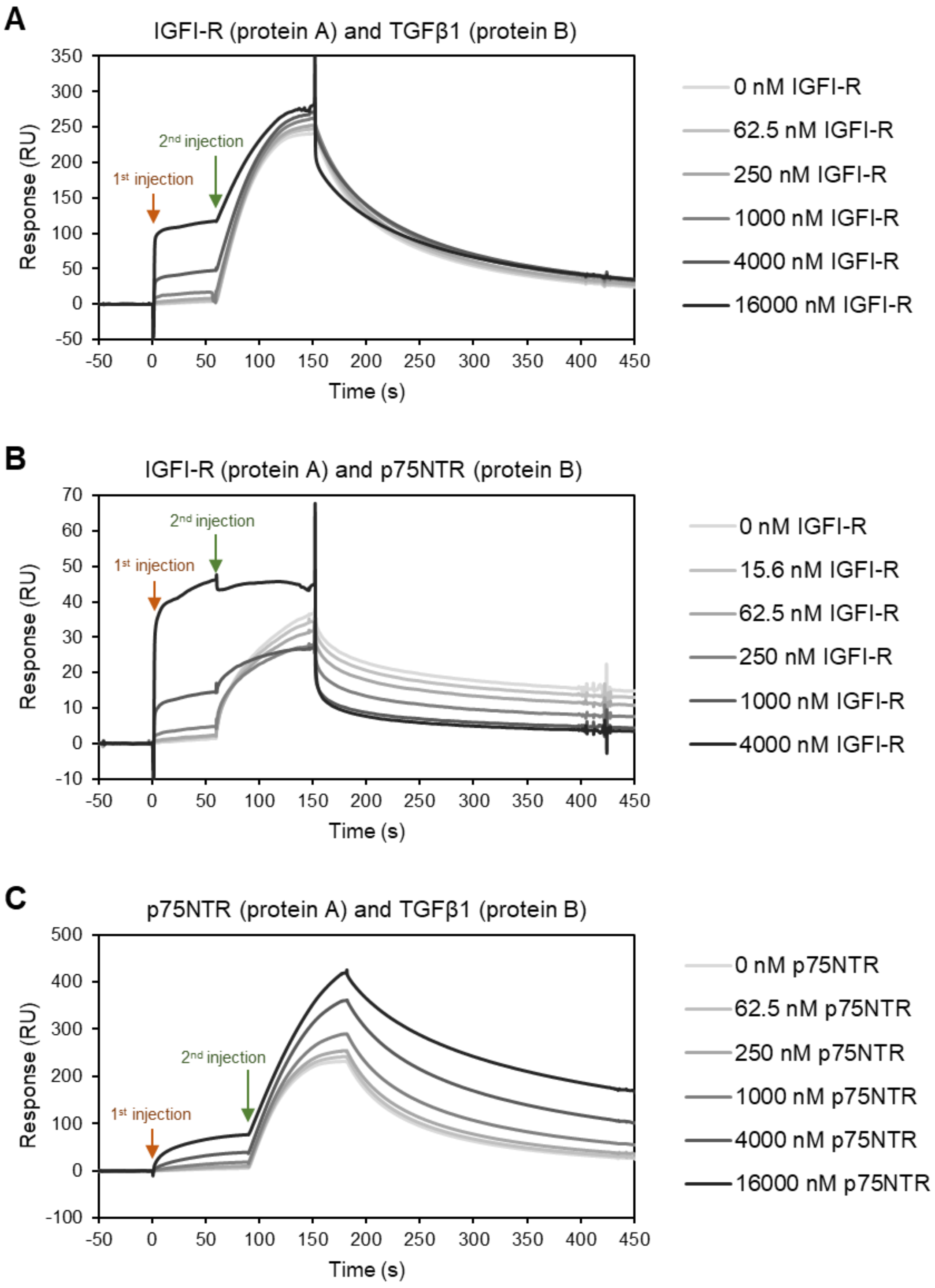


**Figure S7.** SPR raw sensorgrams of dual injection assay. The assay was performed according to the experimental scheme shown in Figure 3A using the following pairs: (A) IGFI-R (protein A) and TGFβ1 (protein B), (B) IGFI-R (protein A) and p75NTR (protein B), and (C) p75NTR (protein A) and TGFβ1 (protein B).

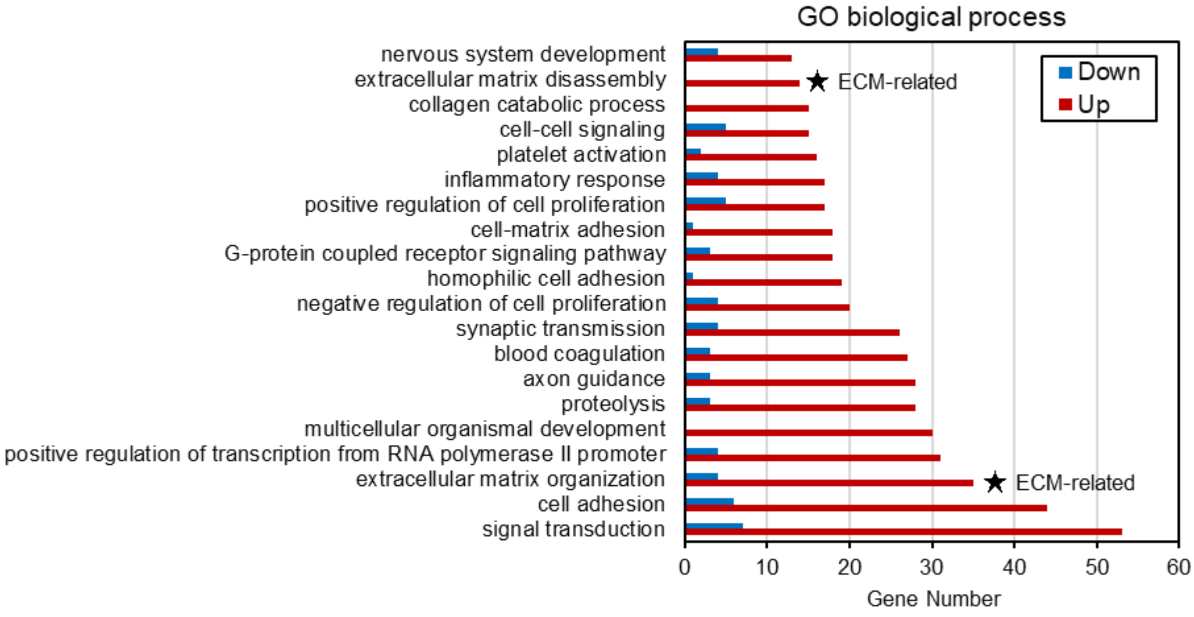


**Figure S8.** GO enrichment histogram. X axis indicates the number of differentially expressed genes in biological process GO term. The top 20 GO terms containing a greater number of up-regulated genes in A549 cells treated with 2,000 nM of the recombinant PRELP are shown.

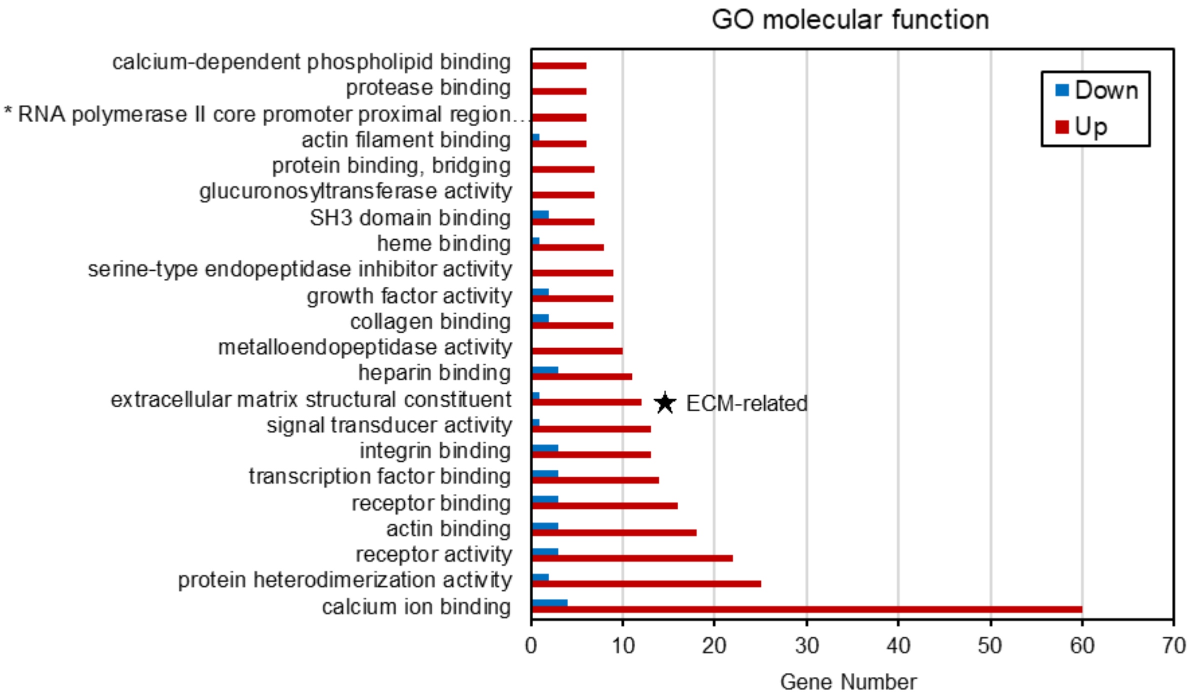


**Figure S9.** GO enrichment histogram. X axis indicates the number of differentially expressed genes in molecular function GO term. The top 20 GO terms containing a greater number of up-regulated genes in A549 cells treated with 2,000 nM of the recombinant PRELP are shown. The category indicated by * is "RNA polymerase II core promoter proximal region sequence-specific DNA binding transcription factor activity involved in positive regulation of transcription."

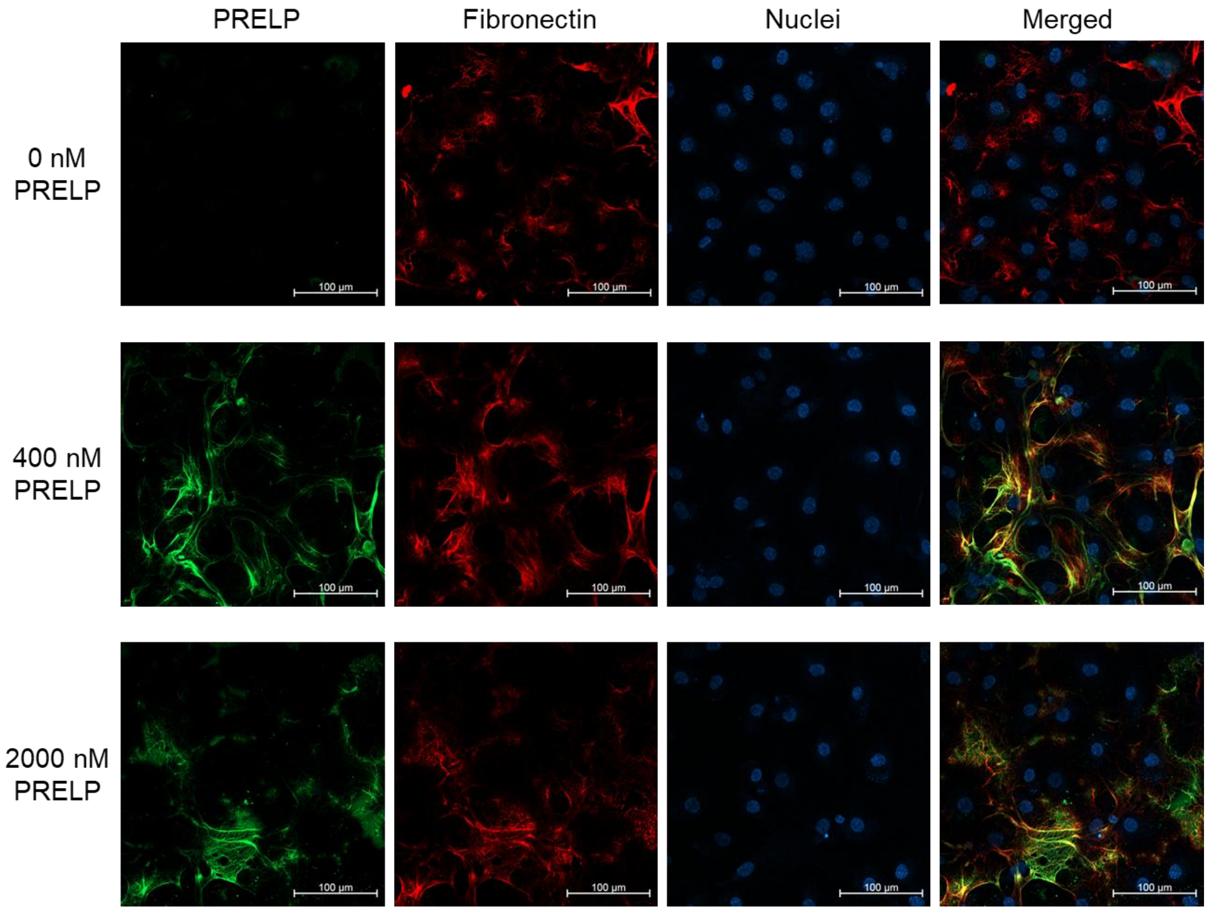


**Figure S10.** Double immunofluorescence staining of PRELP and fibronectin in HUVECs treated with different concentrations (0, 400, and 2,000 nM) of the recombinant PRELP. Nuclei were visualized by DAPI staining. Scale bar: 100 μm.

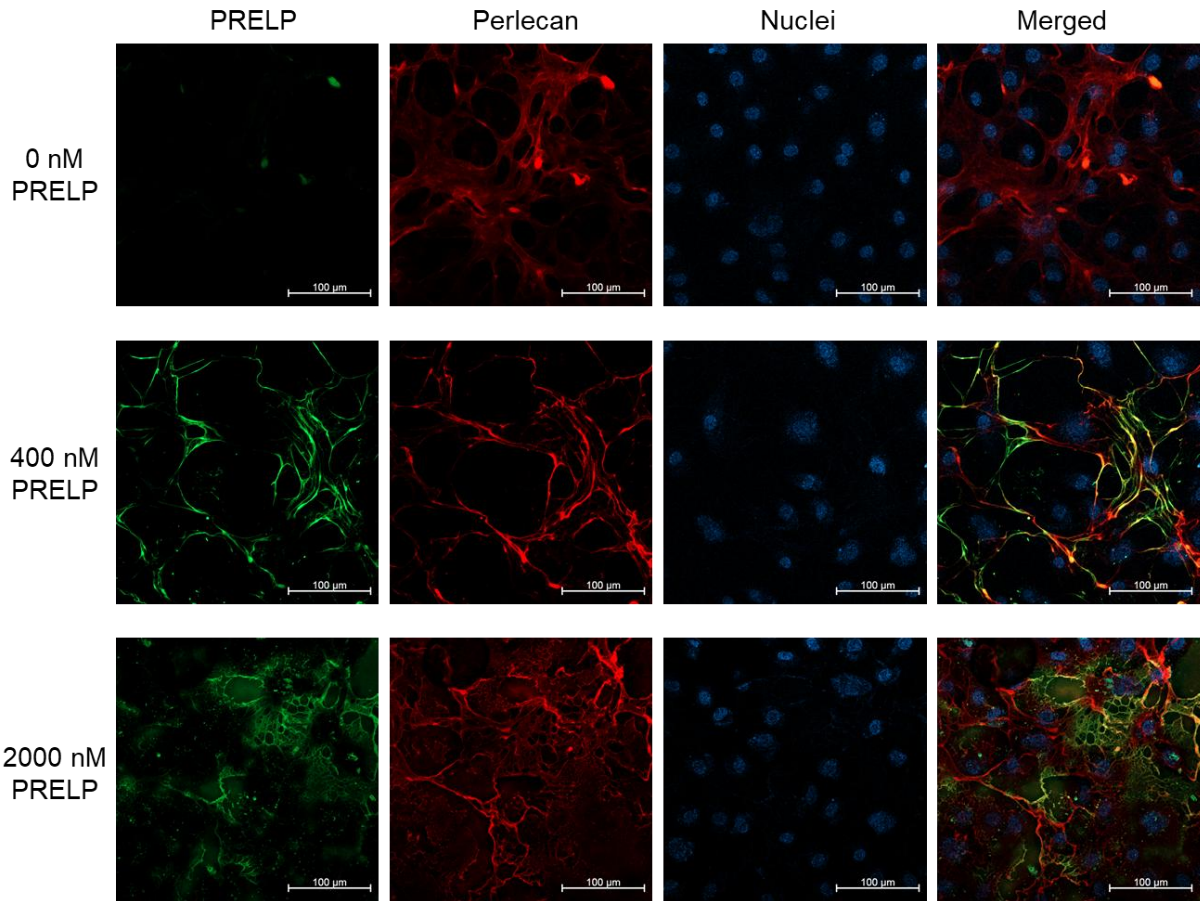


**Figure S11.** Double immunofluorescence staining of PRELP and perlecan in HUVECs treated with different concentrations (0, 400, and 2,000 nM) of the recombinant PRELP. Nuclei were visualized by DAPI staining. Scale bar: 100 µm.

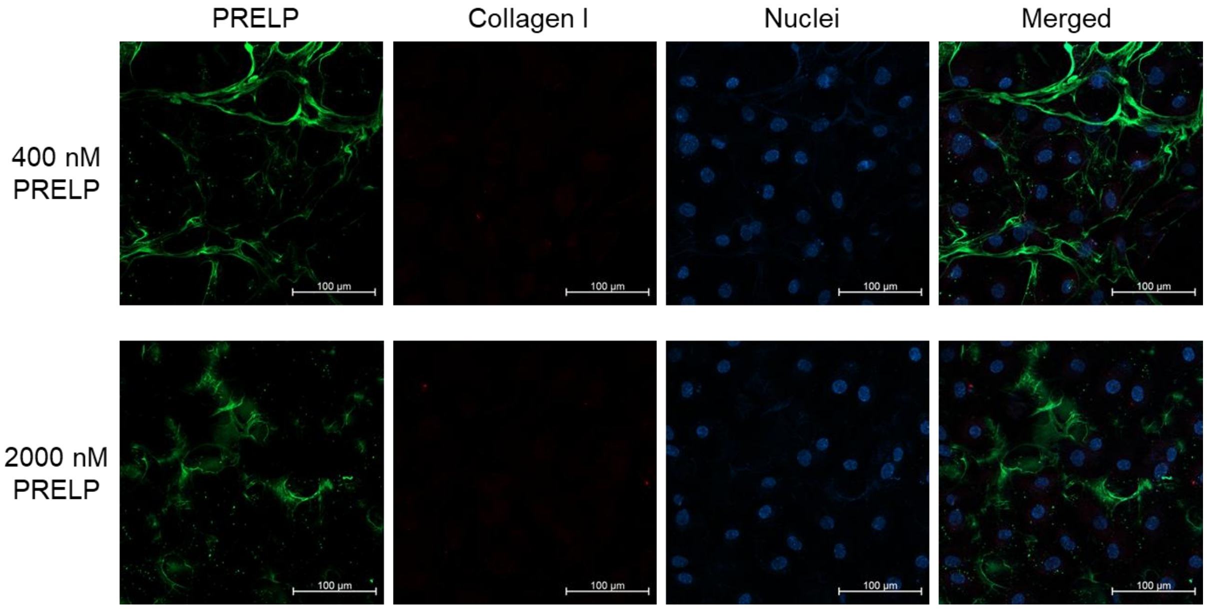


**Figure S12.** Double immunofluorescence staining of PRELP and type I collagen in HUVECs treated with different concentrations (400 and 2,000 nM) of the recombinant PRELP. Nuclei were visualized by DAPI staining. Scale bar: 100 μm.

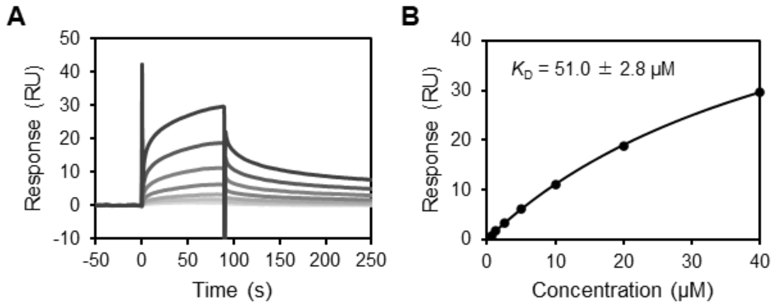


**Figure S13.** Direct binding assay using SPR between p75NTR and TGFβ1. (A) SPR response vs. time curves for the interaction at 15 °C. TGFβ1 was immobilized on a CM5 sensor chip at around 1,400 RU, and p75NTR was injected into the sensor chip in a dose-dependent manner (625–40,000 nM) in 10 mM HEPES (pH 7.5), 150 mM NaCl containing 0.05% (v/v) Tween-20. Line darkness indicates concentration. The darkest line indicates the highest concentration. (B) Response units vs. concentration of p75NTR for the interaction. The $K_D$ value with standard error of curve fitting was calculated: $K_D$ = 51.0 ± 2.8 μM.
